# Stress Management Training (SMT) Improves Coping of Tremor-Boosting Psychosocial Stressors and Depression in Patients with Parkinson's Disease: A Controlled Prospective Study

**DOI:** 10.1155/2018/4240178

**Published:** 2018-10-28

**Authors:** C. Buhmann, D. Jungnickel, E. Lehmann

**Affiliations:** ^1^Department of Neurology, University Medical Center Hamburg-Eppendorf, Martinistrasse 52, 20246 Hamburg, Germany; ^2^Department of Social Sciences, University of Hamburg, Allende-Platz 1, 20146 Hamburg, Germany

## Abstract

**Background:**

Stress reduction and relaxation exercises are therapeutically suggested to patients with Parkinson's disease (PD) and tremor, but data regarding efficacy or preferential methods are missing.

**Objective:**

To investigate the effect of a standardized stress management training (SMT) according to Kaluza on coping with tremor-boosting psychosocial stress factors.

**Methods:**

8-week SMT was applied to 82 PD patients with tremor and 30 controls. Changes in stress-associated factors were measured applying four scales: Kaluza's “warning signs for stress” and “stress-amplifying thoughts” and Beck Depression Inventory (BDI) and quality of life (PDQ-8). Short-term outcome (8 weeks) was evaluated in both groups, and long-term outcome (3–6 months) was evaluated only in PD patients.

**Results:**

At baseline, PDQ-8 was worse in PD patients compared to controls. PD patients improved significantly regarding short- and long-term outcome scores of “warning signs for stress,” “stress-amplifying thoughts,” and BDI scores, independently of disease severity or duration. Younger and male PD patients showed the best benefit. Controls improved comparably to PD patients but significantly only with respect to “stress-amplifying thoughts.” Retrospectively, 88% (29/33) of PD patients were rated SMT as helpful 12–18 months later. Self-practicing SMT exercises correlated significantly with subjectively better coping with tremor-related daily impairment and subjective short-term and long-term tremor reduction.

**Conclusion:**

SMT should be a part of therapy of PD patients with tremor.

## 1. Introduction

Tremor is a frequent symptom in Parkinson's disease (PD) and affects the majority of patients already as an early motor sign [1]. Although medication generally improves tremor in PD to more than 50% [2], a relevant proportion of patients remain disabled by their tremor. In these patients, tremor is not only functional hindering but also clearly visible as a disease symptom. This can lead to real or felt social stigmatization, anxiety, depression, or social isolation [3, 4] and reduced quality of life [5].

Although there are limited data of clinical studies confirming stress-induced tremor in PD [6], it is clinically well known and has been described early in the literature that tremor in patients with PD increases when stress factors or emotional involvement is present [7, 8]. Hereby, the increase of adrenergic activation is supposed to play a key role [9, 10]. In daily clinical practice, patients are suggested to use relaxation techniques to reduce tension and stress levels to improve frequency, intensity, or coping of existing tremor. However, there is lack of clinical data on whether relaxation training really can improve coping with tremor and/or reduce tremor in PD. Relaxation techniques seem to be of different effect [11], but there are no controlled data on which kind of relaxation training might be most effective.

For healthy subjects, it has been shown that stress management programs imparting relaxation skills improve coping of general stress-related tension and stress-related situations [12–15].

Participation in the cognitive-behavioural stress management training (SMT) according to Kaluza [16, 17] has been proven to be efficient for coping of all-day stressors with a change in problem awareness and improvement of emotional and physical well-being [18] based on improved active and cognitive coping strategies and decreased subjective appraisals of interpersonal stressors [19]. SMT was effective not only in healthy subjects but also in patients with chronic diseases other than PD [20]. Furthermore, long-term effects of SMT have been demonstrated for healthy subjects with ongoing improved active coping strategies, less resigned-avoidant tendencies, and improved psychological mood status [21] 6 months after intervention.

In this study, we primarily aimed to evaluate whether participation for 8 weeks in SMT [16, 17] leads to improvement of tremor-boosting stressors, expressed more specifically as warning signs for stress and stress-amplifying thoughts and reflected more generally as depression and quality of life. Secondarily, we assessed the influence of patient characteristics on SMT outcome measurements and evaluated the subjective benefit of SMT in PD patients on coping of tremor-related daily impairment.

We hypothesized a beneficial effect of SMT on stress levels and coping of tremor in PD patients.

## 2. Methods

### 2.1. Study Design

In this monocentric, controlled, and prospective interventional study, 82 PD patients with tremor and 30 controls underwent stress management training as group therapy with 6–8 participants for 2 hours per week for 8 weeks. The influence of the training was evaluated based on the questionnaire at baseline, as short-term outcome at the end of the course after 8 weeks, and as long-term outcome after 3–6 months. In a pilot study with participants from different social demographic sectors, intelligibility of the questionnaire had been evaluated and considered for the final version. Non-PD subjects served as controls for estimation of baseline stress levels in PD patients prior to SMT. Although we primarily did not aim for group comparison, we also analyzed whether SMT had differential effects in PD patients compared to controls after the end of SMT. The course and the evaluation were realized from 2013 until 2016 within the facilities of the University Medical Center Hamburg-Eppendorf (UKE).

First, we assessed as the primary aim prospectively the influence of the stress management training (SMT) as described by Kaluza [16] and specified later [17] on levels of warning signs for stress and stress-amplifying thoughts on depression according to Beck Depression Inventory (BDI) [22, 23] and on quality of life (QoL) according to PDQ-8 [24]. Second, we evaluated the influence of age, gender, disease severity [25], and disease duration on outcome parameters. Subsequently, we retrospectively assessed the influence of the training on self-estimation of tremor-related daily impairment and tremor intensity in a follow-up investigation during a normal clinical consultation 12–18 months after the end of SMT using a 5-point Likert's scale.

Questionnaires had to be completed under identical conditions for each participant prompt and without interruption in presence of the course teacher (EL). Contact with a third party was not allowed. In case of questions for a better understanding or other needs for support to complete the questionnaire, participants were allowed to ask the instruction teacher. Completion of the questionnaire took an average of 1 to 1.5 hours. After completion of the questionnaires, data were pseudonymized for analysis.

### 2.2. Ethics

Written informed consent was obtained from every participant. The research protocol considered the Good Clinical Practice (GOP) criteria, followed the recommendations of the Declaration of Helsinki (7^th^ revision, 64^th^ meeting, Fortaleza, Brazil), and was approved by the Ethics Committee of the Hamburg Medical Council (votum PV 4886).

### 2.3. Patients and Controls

Patients were recruited in the movement disorder outpatient clinic of the University Medical Center Hamburg-Eppendorf. PD diagnosis according to UK Brain Bank criteria [26] was confirmed by a movement disorder specialist (CB). Patients who reported to be psychologically impaired due to tremor (independently of tremor severity) or patients with an objective (physician-assessed) functional tremor-related impairment (such as problems with writing or dexterity) were consecutively asked to take part in the stress management training and enrolled if agreed to take part in the study.

Controls were not allowed to have any present or history of neurological or psychiatric disease or any other relevant acute or chronic disease and were recruited from the familiar or social surrounding of the PD patients or the working staff of the hospital. Subjects with known severe psychosocial impairment interfering with participation in a group therapy were excluded. Furthermore, subjects with relevant cognitive impairment were excluded because they potentially would not have been able to fulfill demands of the course. We chose an MMSE ≤ 20/30 points as cutoff because Folstein et al. found MMSE values of 20 or less only in patients with dementia [27].

### 2.4. Interventional Training Course (Stress Management Training (SMT))

The cognitive-behavioural stress management training (SMT) according to Kaluza [16, 17] was applied to course participants under supervision of a certificated psychologist (EL). The SMT consists of different modules aiming to cope with and reduce stress levels: 4 training modules and 5 supplemental modules supporting activation of positive resources. Training modules consist of (i) relaxation training such as progressive muscle relaxation according to Jacobsen [28] (modified version according to [29]) or autogenic training, (ii) cognition training to detect and modify personal stress triggers or boosters [17], (iii) structured problem-solving training to detect, accept, and modify stress situations, and (iv) enjoyment training for recreation and enjoyment [30, 31]. Supplemental modules included stress management instruments such as physical activity, social support, definition of aims, time management, and emergency strategies.

All patients completed the four training modules and were advised to choose one supplemental module.

### 2.5. Questionnaire-Based Assessment

Two dimensions of psychosocial stress factors according to Kaluza, i.e., “levels of warning signs for stress” and “stress-amplifying thoughts,” and depression and quality of life were assessed.

#### 2.5.1. Psychosocial Stress Factors according to Kaluza

The total score of *warning signs for stress* ranges from 0 to 70 points and is based on 35 symptoms/characteristic in the 4 categories “physical warning signs” (14 questions), “emotional warning signs” (6 questions), “cognitive warning signs” (6 questions), and “behavioural warning signs” (9 questions). Each symptom was self-rated by the patient to be present “barely or not at all” (score 0), “slight” (score 1), or “severe” (score 2). For individual diagnostics, outcome is divided into 3 categories. 0 to 10 points correspond with relative good health stability. In these subjects, relaxing training likely will have a prevention character; 11–20 points indicate that chain reactions of physical and mental stress reactions do take place already. The start of increasing personal competences in stress management is suggested in time; 21 points or more indicate that the subject is already deeply involved into the vicious circle of tension, emotional burden, and health dysfunction. Here, the subject should do something against his/her stress, and it is strictly suggested that the subject has to ensure to get more calmness, rest, and capability [17].  Table 1(A) shows the 35 questions of warning signs in the 4 categories.

The total score of *stress-amplifying thoughts* ranges from 0 to 50 points and is based on the 5 categories “be perfect,” “be popular,” “be strong,” “be on your guard,” and “I cannot.” Each category consists of 5 attributes. Presence of each attribute was rated by the patient to be either 0 (“never”), 1 (“sometimes”), or 2 (“frequent”). The higher the value is, the more the stress-amplifying thoughts in the subdomain or in total the subject has.  Table 1(B) shows the 25 questions of stress-amplifying thoughts in the 5 categories.

#### 2.5.2. Depression and Quality of Life

To evaluate depression, Beck Depression Inventory (BDI) [22, 23] was used. To assess quality of life (QoL), PDQ-8 [24] was used.

### 2.6. Statistics

Analysis was done using SPSS 6.1.3 (1995) for Microsoft Windows. Power analysis was performed using G^*∗*^Power [32] and revealed a sample size of *n* = 80 based on an alpha-failure of 1%, a test power of 90%, and expecting average effects of *d* = 0.35 (absolute).

As known for the PDQ-8, where the 8 items were summed together and transformed onto a score from 0 to 100, scales according to Kaluza (warning signs for stress and stress-amplifying thoughts) and BDI values were converted to POMP scores also for comparison purposes with the range from 0 to 100% [33].

For longitudinal assessment, treatment effects on different scales for the intervention group were calculated using Student's paired *t*-test for dependent groups by calculating the differences in test scores from short term (8 weeks) and long term (3–6 months) versus baseline, respectively. Positive values of calculated differences indicate worsening, and negative values indicate improvement for all applied scales.

Between-group differences of outcome parameters were calculated using Student's *t*-test for independent groups and due to a high number of single subitem comparisons (*n* = 126) applied only for items with Cohen's *d* value >0.25. Data of all applied total scores (BDI, PDQ-8, “warning signs of stress,” and “stress-amplifying thoughts”) were normally distributed (Kolmogorov–Smirnov's goodness-of-fit test).

The chi-squared test and Pearson's *R* test were used to assess the influence of disease severity and disease duration on outcome measurements.

Point-biserial correlations were applied to evaluate potential patient characteristic immanent bias influencing the drop-out rate at long-term evaluation.

## 3. Results

Eighty-two patients with PD and 30 controls completed the stress management training (SMT). Short-term data of prospective outcome measurements were complete at the end of the course after 8 weeks in 82 PD patients (100%) and 27 controls (90%). 3 controls terminated SMT prematurely (2 due to lack of time and 1 reported discomfort in a group with PD patients). Long-term outcome measurement data were complete for the study follow-up examination 3–6 months after the end of the course in 49 PD patients (59.8%; missing subjects could not have been reached or motivated for the follow-up investigation). The drop-out rate was not influenced by age, gender, BDI score, or PDQ-8 score (the range of 2-tailed tested correlations was −0.212 to +0.146 with all correlations being insignificant).

Furthermore, retrospective subsequent outcome data of 33 PD patients (40.2%) could have been obtained at a normal scheduled clinical follow-up consultation to assess the effect of SMT and ongoing self-practice of exercises 12–18 months after the end of SMT. Patients with available long-term data did not differ regarding age, gender, BDI score, and PDQ-8 score from patients without reported long-term data (59.8%). Long-term outcome of controls was not assessed as controls primarily were included to estimate the global level of tremor-boosting psychosocial stress factors in PD patients.

### 3.1. Characteristics of Participants

Parkinson's disease patients were in average 6.4 years older than controls and had a highly significant worse quality of life (25.82 [16.35] vs. 5.77 [9.41]; *p* < 0.0001; *d* values of all PDQ-8 subitems ranged from 0.81 to 1.33) [34]. No significant differences were seen regarding gender or for the aspects depression (total BDI score), warning signs for stress, or stress-amplifying thoughts (total scores and all single items) between PD patients and controls prior to SMT (Cohen's *d* values ranged from −0.28 to 0.31). However, 2 of 21 subitems of the BDI (worthlessness [*d* value 0.45] and loss of energy [*d* value 0.40]) were different between groups at baseline (Table 2).

Within the PD patient group, genders did not differ significantly regarding any outcome measurement parameter at baseline, but women with PD tended to be more depressive than men (BDI score 21.78 [11.3] vs. 17.09 [11.5]; *p*=0.070). Disease severity (H&Y) categorized into mild (H&Y stage 1), moderate (H&Y stage 2), and severe (H&Y stages 3 and 4) did not differ significantly between genders (*χ*^2^ (2,*N* = 82) = 5.05; *p*=0.080). However, while percentage of patients with H&Y stage 2 was almost identical in both genders (55.3% and 56.1%), men were by trend more severely diseased than women with H&Y stage ≥3 in 34% vs. 17%. H&Y stage 4 was only seen in 2 patients (1 male/1 female). It is worth to note that disease severity (H&Y) was not dependent on disease duration (*χ*^2^ (2,*N* = 79) = 3.59; *p*=0.464).

### 3.2. Effect of Stress Management Training (SMT) on Psychosocial Stress Factors according to Kaluza in PD Patients and Controls

SMT significantly improved coping of stress expressed as reduced warning signs of stress and stress-amplifying thoughts as short-term outcome and long-term outcome in PD patients. In controls, significant improvement of stress-amplifying thoughts was obtained as short-term outcome.

**Figure 1:**
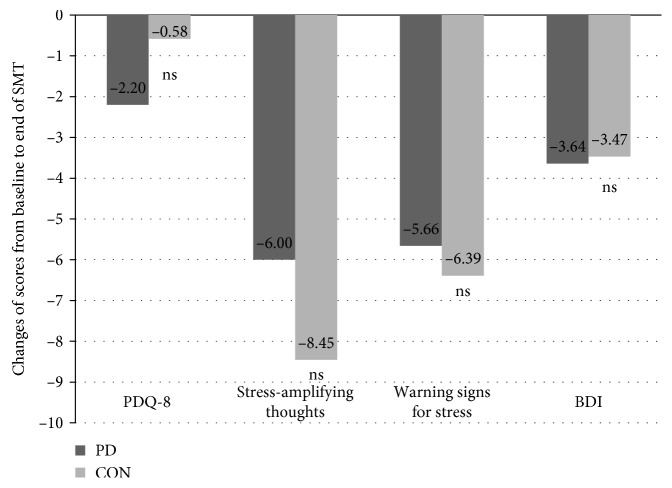
Comparison of changes in psychosocial stress factors according to Kaluza, severity of depression (BDI), and quality of life (PDQ-8) in PD patients and controls after 8 weeks of SMT. The short-term changes in psychosocial stress factors according to Kaluza (warning signs for stress and stress-amplifying thoughts), severity of depression according to Beck Depression Inventory (BDI), and quality of life (PDQ-8) of PD patients and controls from baseline (black bars) at the beginning of stress management training (SMT) to the end of SMT after 8 weeks (light grey bars) are shown. Differences in scale values refer to scales transformed to a score from 0 to 100 points (0–100%) for comparison purposes after conversion to POMP scores [33]. PD = patients with Parkinson's disease; CON = healthy controls.

PD patients and controls did not differ significantly regarding the magnitude of improvement of any psychosocial stress factor at the end of SMT after 8 weeks (Figure 1).

**Figure 2:**
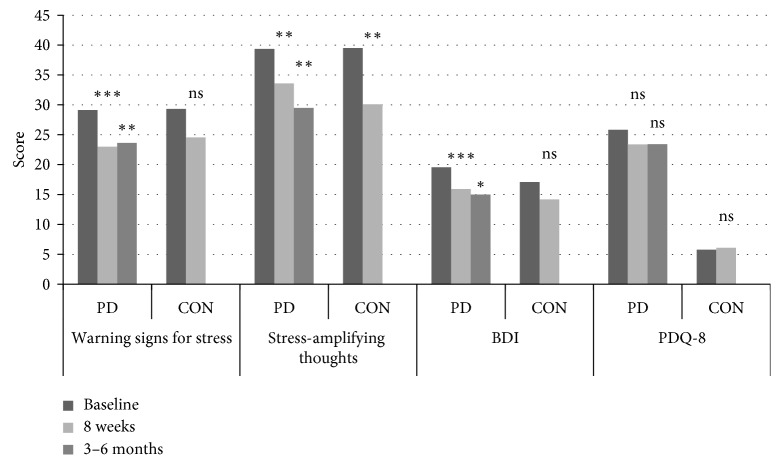
Psychosocial stress factors according to Kaluza, severity of depression (BDI), and quality of life (PDQ-8) of PD patients and controls before and after SMT. Psychosocial stress factors according to Kaluza (warning signs for stress and stress-amplifying thoughts), severity of depression according to Beck Depression Inventory (BDI), and quality of life (PDQ-8) of PD patients and controls at baseline (black bars) at the beginning of stress management training (SMT) and as short-term outcome directly at the end of SMT after 8 weeks (light grey bars) are shown. Outcome of PD patients additionally is shown as long-term outcome 3–6 months after completing SMT (dark grey bars). All scales are summed together and transformed to a score from 0 to 100 points (0–100%) for comparison purposes after conversion to POMP scores [33]. Significantly different scores between baseline and short-term outcome (8 weeks) and baseline and long-term outcome (3–6 months) are labeled with an asterisk with ^*∗*^*p* < 0.05, ^*∗∗*^*p* < 0.01, and  ^*∗∗∗*^*p* < 0.001. PD = patients with Parkinson's disease; CON = healthy controls.

Figure 2 shows an overview, and Tables 3 and 4 show detailed results of outcomes with respect to all total scores and subscales. Subscales of the applied German BDI score [23] were translated into English by the authors.

#### 3.2.1. Short-Term Outcome at the End of the Course after 8 Weeks

In PD patients, SMT improved total mean [±SD] of global scores for warning signs of stress (−5.66 [11.50]; *p* < 0.0001) with all 4 single warning signs (physical, emotional, cognitive, and behavioural) found significantly better at the end of training. Thereby, improvement of emotional stress was most prominent with 28.6%.

Furthermore, total mean [±SD] of stress-amplifying thoughts improved (−6.00 [17.19]; *p*=0.003) with 3 of 5 single stress-amplifying thoughts (“be popular,” “be on guard,” and “I cannot”) getting better. Thereby, the stress factor “to be popular” improved the most with reduction of this demand of 24.0%.

In controls, SMT did not improve total mean of global scores for warning signs of stress significantly. However, 1 of 4 single warning signs (emotional warning) was found to be 34.1% better at the end of the training (*p*=0.014).

SMT improved short-term total mean [±SD] outcome of stress-amplifying thoughts (−8.45 [11.87]; *p*=0.003) in controls with 4 of 5 single aspects (“be perfect,” “be popular”, “be on guard” and “I cannot”) getting better and best improvement for the item “be popular” with 26.1%.

#### 3.2.2. Long-Term Outcome at Follow-Up after 3 to 6 Months

In PD patients, SMT improved total mean [±SD] of global scores for warning signs of stress (−4.86 [11.98]; *p*=0.008), including 2 of 4 single warning signs (emotional and cognitive). It is worth to note that improvement of emotional stress compared to baseline was maintained on the high level with 30.2%.

Total mean [±SD] of stress-amplifying thoughts improved (−7.71 [16.52]; *p*=0.002), including 2 of 5 single stress-amplifying thoughts (“be popular” and “I cannot”). Thereby, the burden to think “I cannot” even improved from 22.8% after the end of the course to 39.0% in the long term 3–6 months later.

### 3.3. Effect of Stress Management Training (SMT) on Depression and Quality of Life in PD Patients and Controls

SMT significantly improved depression assessed as the short-term and long-term BDI outcome in PD patients. Improvement of the BDI score did not reach significance after the end of SMT in controls, although magnitude of improvement was comparable in both PD patients and controls (Figure 1). Quality of life was by trend better directly after the end of SMT in PD patients (PDQ-8 sum score −2.20 [11.14]; *p*=0.082) but without reaching statistical significance for both short- and long-term outcomes.

#### 3.3.1. Short-Term Outcome at the End of the Course after 8 Weeks

In PD patients, SMT improved total mean [±SD] of the BDI score (−3.64 [8.26]; *p* < 0.0001). Amongst the 21 subscales of the BDI scale, 8 significantly improved (Table 3). Hereby, “attachment disorder” (*p* < 0.0001), “feeling of guilt” (*p*=0.003), and “indecisiveness” (*p*=0.010) were improved most markedly with 36.3%, 49.2%, and 29.0%, respectively.

In controls, no significant change in the total mean BDI score was seen. However, 2 of 21 subscales improved significantly (change in “sleep disturbance” (*p*=0.006) and “attachment disorder” (*p*=0.036), with 36.3% and 40.0%, respectively; Table 3).

#### 3.3.2. Long-Term Outcome at Follow-Up after 3 to 6 Months

In PD patients, SMT improved outcome of total mean [±SD] of the BDI score (−3.21 [8.77]; *p*=0.014). Thereby, the 3 subitems “feeling inadequate/inferior” (*p*=0.049), “attachment disorder” (*p*=0.31), and “suicidal contents” (*p*=0.10) improved about 65.7%, 20.0%, and 62.2%, respectively.

### 3.4. Influence of Age, Gender, Disease Severity, and Disease Duration on the Efficacy of the Stress Management Training (SMT) on Psychosocial Stress Factors in PD Patients

With higher age, efficacy of SMT on reduction of depression was lower in the short-term (*r* = 0.30; *p* < 0.01) and long-term (*r* = 0.29; *p* < 0.01) follow-up. Age did not influence any other measured outcome parameter. Despite that, subjective efficiency of SMT was rated higher in the younger patient (*r* = −0.35; *p* < 0.01).

In men, benefit of SMT on long-term outcome of BDI (categorized into better, unchanged, and worse) was better than that in women (*χ*^2^ (2,*N* = 82) =11.83 (*p*=0.003) and *r* = 0.33 (*p*=0.003)). Gender had no influence on short-term or long-term outcomes for warning signs of stress, stress-amplifying thoughts, or quality of life.

Neither disease severity nor disease duration had any influence on short- or long-term outcomes of any measured coping factors after SMT.

### 3.5. Subjective Benefit of SMT in PD Patients on Coping of Tremor-Related Daily Impairment in General and in Relation to Tremor Reduction and Self-Exercising

Twelve to 18 months after finishing SMT, patients were asked during a regular consultation how they retrospectively rate the SMT regarding a general benefit on coping tremor-related daily impairment (yes/no) and the effect on subjective tremor reduction in daily life at the end of the course (8 weeks) and 3–6 months after SMT and whether they self-practiced the SMT at home beyond 3 months after the end of the course (yes/no).

Answers could have been obtained from 40% of PD patients (33/82). 87.9% (29/33) rated the SMT as beneficial in general for coping with tremor in daily life, independently of gender. General benefit of SMT on life was more reported by patients who self-practiced exercises at home (*χ*^2^ (1,*N* = 26 (reduced *N* according to missing data)) = 5.52; *p*=0.019; and *r* = 0.461; *p*=0.018) and who reported subjective reduction of tremor at the end of the SMT course (*χ*^2^ (1,*N* = 32) = 5.88; *p*=0.015; and *r* = 0.429; *p*=0.015). Ongoing home exercising correlated with tremor reduction 4 weeks (*χ*^2^ (1,*N* = 25) = 4.57; *p*=0.033; and *r* = 0.428; *p*=0.033) and >3 months (*χ*^2^ (1,*N* = 24) = 10.15; *p*=0.0014; and *r* = 0.650; *p*=0.0006) after SMT.

## 4. Discussion

It is well known that tremor in patients with PD increases with stress factors or emotions. The objective of this study was to evaluate prospectively the effect of the structured educational stress management training (SMT) that teaches techniques for physical and mental stress reduction on coping of tremor-boosting psychosocial stressors according to Kaluza, depression, and quality of life in PD patients with tremor. We did not aim to evaluate the effect of SMT on objectively measured tremor or to compare SMT effects between PD patients and controls. Controls served as an indicator for baseline stress levels in PD patients prior to SMT and were used to assess whether SMT might be especially effective in PD patients with tremor compared to other subjects.

We showed that SMT reduces tremor-boosting psychosocial stressors such as warning signs of stress and stress-amplifying thoughts as well as depression independently of disease severity and disease duration in the short-term (8 weeks) and long-term (3–6 months) follow-up in PD patients.

Both PD patients and controls benefit comparably from SMT after 8 weeks of training. While improvement of stress-amplifying thoughts was significant in both subject groups, improvement of warning signs of stress and depression reached statistical significance only in the PD group. Within the PD group, improvement of depression was better in younger patients and in men. The majority of patients rated SMT retrospectively as beneficial for coping tremor-related daily impairment and self-practicing of SMT exercises at home correlated with subjective tremor reduction.

Emotional stress increases tremor in PD patients [7], and cognitive stress recently has been found to even reduce the levodopa effect [35]. Therefore, it is reasonable that physicians advise their patients to learn and practice relaxation techniques such as progressive muscle relaxation or autogenic training. However, data are lacking on which technique might be preferential to improve tremor intensity or coping with tremor [11].

Our results first support that applying relaxation techniques indeed is meaningful to compensate with tremor-related stress and second suggest that SMT according to Kaluza [16, 17] is helpful for PD patients with tremor. SMT offers a broad approach of stress reduction techniques to improve relaxation, cognitive behavioural aspects, problem solving, and enjoyment. In contrast to other stress management programs, SMT according to Kaluza does not only focus on assessment of negative health conditions such as anxiety or depression but also considers outcome measurements of positive aspects such as awareness of stressors, emotional well-being, and subjective changes of burden or coping strategies [36], which are primary measures for health promotion. The training has been proven to increase active and cognitive coping strategies, to improve mood states, and to decrease subjective appraisals of interpersonal stressors in the family and at work in a study with 99 healthy subjects [19]. PD patients with tremor suffer even more from psychosocial stressors due to their physical dysfunction and visible impairment [3, 4]. As a vicious circle, increased stress and emotional involvement increase tremor, and vice versa [7]. As a result, it is rather the acute stressful situation such as searching for coins in the purse at the supermarket cash desk or eating in a nonfamiliar society that triggers tremor than a general increased stress level. Most PD patients report that they have much less tremor in their familiar setting. This is supported by our results showing comparable baseline psychosocial stress levels between PD patients and controls and a comparable improvement after SMT. It is worth to note that controls were subjects without history of any relevant disease but not necessarily individuals without elevated stress levels and/or mild depressive symptoms. In contrast to PD patients, controls indeed had a mean baseline BDI score (17 points) still in the range defined as normal and below the mean cutoff defined to express mild depression in the original work of Beck [37]. However, especially standard deviation of 11 points and suggested lower BDI screening cutoffs for mild depression in a general population (e.g., 12/13 points) [38] indicate that some controls exhibited depressive symptoms. Part of controls was related to PD patients, which might trigger depressive symptoms [39].

Nevertheless, QoL in PD patients was worse compared to that in controls prior to and at the end of SMT, indicating additional other problems than tremor in PD patients.

In PD patients, improvement of self-perception of stressors and warning signs for stress and development of stress management strategies are meaningful to break the vicious cycle in tremor-boosting situations. In accordance, SMT especially decreased the items “emotional stress” and the stressful thoughts to “be popular” and “I cannot” in PD patients. These aspects are crucial to keep calm in stressful situations. The results indicate that patients acquired better self-confidence with less negative disease-related emotional involvement. Additionally, as other relaxation trainings, SMT offers PD patients a possibility for a general improvement of the well-being, and participation in the course was rated to be beneficial and pleasant by most of the patients.

Although patients' motivation for change and their perceived self-efficacy are not well studied to date [40], we demonstrated that PD patients were able and willing to learn stress management techniques for 2 hours weekly over 8 weeks and accept SMT as chance to lower disease-related stress factors and symptoms. In accordance, none of the PD patients finished SMT prematurely (in contrast to controls). Improvement of psychosocial stressors persisted at least up to 3–6 months after SMT, suggesting long-term coping with stressors is possible. However, ongoing self-exercising seems to be necessary to maintain improved stress management strategies in PD patients. This probably differs in healthy subjects, where maintained active and distancing cognitive coping, compensation strategies, less resignative-avoidant tendencies, and improved psychological mood states have been found 6 months after termination of SMT in the majority of participants as compared to a noninterventional control group [21]. This difference likely is PD immanent. As known for other nonmedical interventions with demand of regular physical activity, treatment effects in PD patients are vanishing after stopping active intervention [41]. It has been shown that PD patients perform better with coaching [41], and it is a particular challenge to motivate the patients to self-exercise at home. This assumingly is related to the high prevalence of dysexecutive function independent of dementia [42], apathy [43], fatigue [44], and depression [45] in PD patients.

We suggest that a stress management programme or course should become a brick in the multimodal landscape of activating therapies in treatment of PD patients with tremor. Based on former results of SMT in healthy subjects and patients with chronic diseases [19, 20, 36] as well as on findings in the present study, we recommend SMT as a suitable technique that should be offered by a psychologist or a certified trainer qualified for prevention stress management. We furthermore propose to monitor therapeutic effects applying both stress scales of Kaluza, which have been developed to assess psychosocial aspects. Meanwhile, SMT is part of our therapeutic landscape for PD patients and supported by almost all public health insurances in Germany, who reimburse 80% of the costs and appreciate SMT as a tremor prevention course. However, SMT might be beneficial also in PD patients without tremor. It has been shown that autogenic training, when used as an adjunct to physiotherapy, is more effective than physiotherapy alone in improving motor performances assessed as the UPDRS motor score in PD patients [46]. Relaxation-guided imagery techniques reduced not only tremor [11] but also motor fluctuations in PD [47].

We also found a positive short- and long-term effect of SMT on depression in PD patients. This might be related to less emotional involvement and better coping of tremor. Appropriately, subitems of the BDI improved significantly, which could be related to tremor-associated social stigmatization and isolation, such as feeling inadequate/inferior or self-denial/dislike. However, SMT includes aspects of a cognitive-behaviour therapy (CBT) that has been shown to be effective in a small case series of three depressed PD patients [48] and is an established, evidence-based therapy of depression in other populations [49]. In line, BDI scores improved comparably in controls as short-term outcome. Furthermore, it cannot be excluded that depression improved due to the attention of the therapist or a secure feeling within the group. Of note, depression improved especially in younger and male patients, indicating that also men accept this kind of “psychological” SMT.

Quality of life assessed with the PDQ-8 score was not significantly changed after participation in SMT. However, total PDQ-8 consists of several aspects that cannot be aimed to improve by SMT such as mobility, concentration, communication, or pain. Despite that, there was a trend to an improvement (*p*=0.082) of total PDQ-8 as a consequence of improvements of the subscales “emotional well-being” (significant; *p*=0.046) and “stigma” (by trend; *p*=0.097), supporting efficacy of SMT on depression and coping of tremor.

### 4.1. Study Limitations

Short-term outcome results after 8 weeks of SMT are based on a 100% participation rate in PD patients and 90% in controls. Long-term outcome results 3–6 weeks after SMT were available in only 60% of PD patients and so have to be interpreted with caution.

Both Kaluza's scales to measure psychosocial stressors have not been validated in a validation study. However, both scales have been proven to be sufficient in clinical daily practice, indicating a reliable content validity. Furthermore, 2-factor analysis revealed both a high reliability (Cronbach's alpha coefficient of around 0.90 for homogeneity) and a high factorial validity (for all 9 subscales (4 warning signs + 5 stress-amplifying thoughts)) at baseline with an explained variance of 76% for the two factors of both applied psychosocial scales, suggesting applied questionnaires to be sufficient, reliable, and valid.

The study is not appropriate to evaluate the effect of SMT on tremor severity or frequency. Instead, we assessed as additionally subsequent outcome parameter the efficacy of SMT on tremor coping and self-estimation of tremor severity in daily life. These data were acquired retrospectively and unsystematically during a normal consultation 12 to 18 months after finishing SMT. This implies risk for a recall bias, and only 40% of PD patients participating in SMT could be reached for this assessment.

So these results can only indicate that ongoing self-practice of relaxation techniques is helpful to maintain acquired improved coping with psychosocial stress factors and to perceive tremor as less severe. Prospective further studies should confirm this impression.

To cover the important aspect of quality of life (QoL), we evaluated the effect of SMT on PDQ-8. This instrument has been designed for use in PD patients but not in healthy controls or non-PD populations. Therefore, the significant and high difference of QoL between PD patients and controls found prior to and at the end of SMT might be biased by applying the score also in non-PD subjects. However, due to the enormous difference in PDQ-8 values between groups, it is likely that PD patients indeed have a worse QoL. However, better (but not significantly different) improvement of QoL after SMT compared to controls might be related to the higher scores at baseline in PD patients.

### 4.2. Study Strengths

We demonstrated efficacy of a defined SMT on improvement of physical and mental stressors and depression in a controlled and prospective study in a large group of 82 PD patients with tremor as short-term and long-term outcomes. Intensity of the training course was high with a total of 16 hours, and techniques were taught by a specialized psychologist. To our knowledge, a comparable study concept of applying any stress reduction technique in patients with PD has not been reported so far.

In conclusion, stress management training can help patients to cope with tremor and is suggested as part of the therapeutic concept in PD patients with tremor.

## Figures and Tables

**Table 1:** Psychosocial stress factors according to Kaluza.

(A) Warning signs for stress	
The following aspects can be signs of excessive demand. Which of them did you self-percept within the last week?	
Physical warning signs	Palpitation; tight feelings in the breast; difficulty breathing; difficulty falling asleep; chronic fatigue; indigestion; stomach pain; anorexia; sexual dysfunction; muscle tension; headache; back pain; cold feet/hands; severe sweating
Emotional warning signs	Nervousness/inner restlessness; irritability/anger feelings; fear emotions/fear of failure; dissatisfaction/unbalance; listlessness (including sexual); inner emptiness/“burned out”
Cognitive warning signs	Constantly circling thoughts/musing; concentration disorders; emptiness in the head/“black out”; daydreams; nightmares; loss of power/high frequency of mistakes
Behavioural warning signs	Aggressive behaviour against others/“to fly off the handle”; finger thrumming/to shuffle one's feet/trembling/to gnash one's teeth; speak too fast/stuttering; interrupt someone/inability to listen to someone; irregular eating; consuming of alcohol or drugs for calming; not cultivating private contacts/“to let contacts slide”; smoking more than intended; doing less sport and exercise than desired

(B) Stress-amplifying thoughts	
Personal stress enhancer profile	
I cannot	I cannot stand it; I will fail; I can never make it; I cannot stand the pressure (fear, pain, etc.); problems and difficulties simply are terrible
Be on your guard	It is horrible if something is not running as I want; it is important that I have everything under control; in decisions I must be 100% sure; I always have to think about what could happen; it is terrible if I do not know what is coming towards me
Be strong	I like to do everything myself; strong people need no help; when I rely on others I'm abandoned; without me nothing works; it is terrible to depend on others
Be popular	I do not want to disappoint others; it is terrible when others are evil to me; I want to get along well with all others; It's bad when others criticize me; it is important that everyone likes me
Be perfect	It is not acceptable if I am not able to cope with a job or meet a deadline; I always have to be available for my company; there is nothing worse than making mistakes; there must be 100% reliability on me; I always have to make everything right

Part A shows the 35 questions of warning signs for stress in the 4 categories according to Kaluza [17]. The total score ranges from 0 to 70 points, and each symptom was self-rated by the patient to be present “barely or not at all” (score 0), “slight” (score 1), or “severe” (score 2). The higher the value is, the more the warning signs for stress in the subdomain or in total the subject has. Part B shows the 25 questions of stress-amplifying thoughts in the 5 categories according to Kaluza [17]. The total score ranges from 0 to 50 points, and presence of each attribute was rated by the patient to be either “never” (score 0), “sometimes” (score 1), or “frequently” (score 2). The higher the value is, the more the stress-amplifying thoughts in the subdomain or in total the subject has.

**Table 2:** Characteristics of participants at baseline prior to SMT.

	PD (*n* = 82)	CON (*n* = 30)	*p* value	Test
Age [SD]	66.3 [8.16]	59.9 [15.8]	0.041	*t*‐test
Males (%)	39 (47.6 %)	12 (40 %)	0.48	Phi 0.067
BDI score [SD]	19.55 [11.72]	17.09 [10.99]	0.319	*t*‐test
PDQ-8 [SD]	25.82 [16.35]	5.77 (*n* = 24) [9.41]	<0.0001^*∗*^	*t*‐test
Warning signs for stress [SD]	29.13 (*n* = 80) [17.51]	29.31 (*n* = 29) [16.70]	0.961	*t*‐test
Stress-amplifying thoughts [SD]	39.36 (*n* = 81) [22.72]	39.52 (*n* = 29) [18.73]	0.973	*t*‐test
Disease duration (months) [SD]	5.96 (*n* = 79) [0.51], range 1–23	NA	NA	
Disease severity (Hoehn and Yahr) [SD]	2.15 (*n* = 79) [0.08], range 1–4	NA	NA	

Data are shown as means with standard deviation [SD]. Significantly different frequencies between groups as detected in an unpaired 2-tailed *t*-test with *p* < 0.05 are labeled with an asterisk. “BDI”: Beck Depression Inventory [22, 23]; “PDQ-8”: Parkinson's Disease Questionnaire with 8 questions [24].

**Table 3:** Beck Depression Inventory (BDI).

PD patients (*n* = 82)																				Controls (*n* = 30)										
Scale with subitems	Baseline			Short term (8 weeks)			Long term (3–6 months)			Short-term mean (8 weeks) − baseline mean (paired 2-tailed *t*-test)					Long-term mean (3–6 months) − baseline mean (paired 2-tailed *t*-test)					Baseline			Short term (8 weeks)			Short-term mean (8 weeks) − baseline mean (paired 2-tailed *t*-test)				
	*n*	Mean	SD	*n*	Mean	SD	*n*	Mean	SD	Diff.	*t* value	*df*	*p*(*t*)	Sig.	Diff.	*t* value	*df*	*p*(*t*)	Sig.	*n*	Mean	SD	*n*	Mean	SD	Diff.	*t* value	*df*	*p*(*t*)	Sig.
Sadness/unhappiness	82	14.63	25.17	81	8.23	17.90	48	12.50	23.44	−6.58	−2.49	80	0.015	^*∗*^	−1.39	−0.35	47	0.728		30	14.44	18.94	27	12.35	22.92	−2.47	−0.63	26	0.537	
Pessimism/hopelessness	82	16.67	31.54	81	14.40	31.15	48	16.67	33.69	−2.47	−0.70	80	0.489		−4.17	−0.68	47	0.503		30	7.78	18.94	27	7.41	21.35	−1.23	−0.24	26	0.802	
Feeling inadequate/inferior	82	10.57	20.87	80	5.42	15.41	48	4.86	15.36	−5.42	−2.40	79	0.019	^*∗*^	−6.94	−2.02	47	0.049	^*∗*^	30	8.89	17.36	27	8.64	19.81	0.00				
Inability to enjoy	82	27.24	19.69	81	17.28	17.57	49	19.73	17.90	−9.88	−4.29	80	0.000	^*∗∗∗*^	−5.44	−2.22	48	0.031	^*∗*^	30	27.78	19.74	27	18.52	23.27	−11.11	−2.21	26	0.036	^*∗*^
Conscious guilt	82	14.23	22.24	81	7.41	14.91	48	8.33	16.13	−7.00	−3.01	80	0.003	^*∗∗*^	−2.08	−0.68	47	0.497		30	18.89	22.63	27	13.58	16.69	−6.17	−1.55	26	0.134	
Self-punishment	82	8.54	18.00	81	7.00	13.66	48	3.47	10.29	−1.65	−0.78	80	0.436		−2.08	−1.00	47	0.322		30	5.56	15.37	26	1.28	6.54	−5.13	−1.69	25	0.103	
Self-denial/dislike	82	11.79	19.86	81	7.41	17.48	48	5.56	14.31	−4.53	−2.36	80	0.021	^*∗*^	−4.17	−1.63	47	0.110		30	7.78	14.34	26	8.97	17.78	1.28	0.33	25	0.746	
Self-accusation	82	18.70	30.13	81	16.05	27.44	48	7.64	14.16	−2.88	−0.84	80	0.403		−6.25	−1.77	47	0.083		30	13.33	24.13	26	11.54	18.72	2.56	0.70	25	0.490	
Suicidal contents	82	8.94	15.76	81	5.76	12.68	48	2.78	9.31	−3.29	−2.37	80	0.020	^*∗*^	−5.56	−2.69	47	0.010	^*∗∗*^	30	5.56	15.37	26	3.85	10.86	−2.56	−0.81	25	0.425	
Frequency of crying	82	19.51	28.18	81	14.40	24.69	48	11.11	23.15	−5.35	−1.53	80	0.129		−7.64	−1.56	47	0.125		30	14.44	27.24	26	8.97	22.23	−7.69	−1.66	25	0.110	
Irritability	82	26.02	27.23	80	26.67	32.87	48	15.97	26.62	.42	0.10	79	0.917		−7.64	−1.38	47	0.175		30	27.78	31.66	27	18.52	23.27	−9.88	−1.69	26	0.103	
Indifference to other people	82	11.79	20.54	81	9.05	18.27	49	10.88	17.20	−2.88	−1.09	80	0.277		−0.68	−0.22	48	0.830		30	12.22	20.50	27	11.11	20.67	−2.47	−0.53	26	0.602	
Indecisiveness	82	25.20	23.75	82	17.89	19.73	49	21.09	21.18	−7.32	−2.64	81	0.010	^*∗∗*^	−2.72	−0.94	48	0.351		30	21.11	20.50	26	17.95	19.39	−6.41	−1.55	25	0.134	
Reduced physical appearance	82	29.27	31.60	81	25.93	30.28	48	20.83	30.46	−3.70	−1.05	80	0.295		−3.47	−0.71	47	0.481		30	15.56	27.31	27	13.58	24.91	−1.23	−0.23	26	0.823	
Loss of drive/workableness	82	32.11	26.94	81	24.69	23.44	49	27.21	24.22	−7.82	−2.56	80	0.012	^*∗*^	−0.68	−0.18	48	0.855		30	22.22	18.22	27	27.16	22.72	4.94	1.16	26	0.256	
Sleep disturbances	82	41.46	32.53	81	36.21	29.91	49	36.05	29.53	−5.76	−1.67	80	0.099		−5.44	−1.43	48	0.159		30	47.78	33.54	27	33.33	30.66	−17.28	−3.02	26	0.006	^*∗∗*^
Fatigability/tiredness	82	31.71	20.89	82	29.27	13.24	49	28.57	15.21	−2.44	−0.97	81	0.333		−2.72	−0.81	48	0.420		30	28.89	25.87	26	21.79	18.72	−7.69	−1.24	25	0.228	
Loss of appetite	82	8.13	15.33	80	8.75	18.18	48	6.94	15.31	.42	0.23	79	0.820		−1.39	−0.47	47	0.642		30	8.89	19.44	26	7.69	17.15	−2.56	−1.00	25	0.327	
Loss of weight	82	1.63	11.60	81	0.82	7.41	48	0.69	4.81	−.82	−0.53	80	0.596		−2.08	−0.90	47	0.371		30	0.00	0.00	26	0.00	0.00	0.00				
Somatic preoccupation	82	22.76	22.15	81	27.57	24.03	48	27.08	23.48	4.53	1.44	80	0.153		7.64	1.91	47	0.062		30	21.11	22.29	27	17.28	25.10	−2.47	−0.46	26	0.646	
Loss of libido	82	29.67	37.04	82	27.64	35.06	49	29.93	36.80	−2.03	−0.62	81	0.545		−2.72	−0.66	48	0.511		30	28.89	33.60	27	37.04	37.36	4.94	0.60	27	0.566	
*Ø Beck Depression Score*	82	19.55	11.72	82	15.91	10.81	49	15.00	10.32	−3.64	−3.99	81	0.000	^*∗∗∗*^	−3.21	−2.52	48	0.014	^*∗*^	30	17.09	10.99	27	14.17	10.08	−3.47	−1.61	27	0.119	

^*∗*^ *p* < 0.05; ^*∗∗*^*p* < 0.01; ^*∗∗∗*^*p* < 0.001.

**Table 4:** PDQ-8, stress-amplifying thoughts, and warning signs for stress.

PD patients (*n* = 82)																				Controls (*n* = 30)										
Scales with subitems	Baseline			Short term (8 weeks)			Long term (3–6 months)			Short-term mean (8 weeks) − baseline mean (paired 2-tailed *t*-test)					Long-term mean (3–6 months) − baseline mean (paired 2-tailed *t*-test)					Baseline			Short term (8 weeks)			Short-term mean (8 weeks) − baseline mean (paired 2-tailed *t*-test)				
	*n*	Mean	SD	*n*	Mean	SD	*n*	Mean	SD	Diff.	*t* value	*df*	*p*(*t*)	Sig.	Diff.	*t* value	*df*	*p*(*t*)	Sig.	*n*	Mean	SD	*n*	Mean	SD	Diff.	*t* value	*df*	*p*(*t*)	Sig.
Mobility	82	22.56	20.75	80	20.97	21.18	48	22.57	21.69	−1.32	−0.86	78	0.392		−0.75	−0.42	47	0.676		24	4.75	8.65	19	5.85	10.39	0.46	0.57	17	0.579	
Activities of daily living	82	21.34	18.14	80	21.25	18.09	48	20.66	18.77	0.00					0.78	0.44	47	0.659		24	2.43	11.05	19	3.29	13.36	0.23	1.00	17	0.331	
Emotional well-being	82	30.44	23.21	80	26.56	18.79	48	27.26	19.01	−3.65	−2.02	79	0.046	^*∗*^	−4.60	−2.00	47	0.051		24	6.60	11.32	19	9.21	16.23	1.62	0.72	17	0.484	
Stigma	82	24.01	23.40	80	20.08	17.78	48	16.93	16.46	−3.36	−1.68	79	0.097		−3.26	−1.18	47	0.242		24	4.69	10.14	19	4.61	12.65	−0.69	−0.46	17	0.651	
Social support	82	15.96	20.12	80	14.37	19.67	48	15.28	19.25	−1.67	−0.92	79	0.359		−0.69	−.23	47	0.816		24	1.39	5.31	19	1.75	5.94	0.00				
Cognition	82	33.92	22.29	80	31.41	19.84	48	29.69	19.01	−2.27	−1.16	79	0.251		−2.21	−0.81	47	0.421		24	11.72	19.79	19	11.18	16.35	−2.08	−1.06	17	0.302	
Communication	82	24.29	20.46	80	22.08	19.08	48	21.18	18.11	−1.88	−0.96	79	0.341		−2.78	−1.03	47	0.307		24	2.78	9.73	19	3.51	9.34	0.00				
Bodily discomfort	82	34.04	25.97	80	30.42	22.15	48	33.85	21.91	−3.44	−1.42	79	0.159		0.00					24	11.81	21.41	19	9.21	16.41	−4.17	−1.84	17	0.083	
*Ø PDQ-8 score*	82	25.82	16.35	80	23.39	14.03	48	23.43	13.30	−2.20	−1.76	79	0.082		−1.69	−1.18	47	0.244		24	5.77	9.41	19	6.08	10.27	−0.58	−1.42	17	0.174	
Be perfect	82	36.83	29.48	79	34.30	26.97	48	25.83	24.91	−1.90	−0.65	78	0.515		−6.88	−1.82	47	0.074		30	37.00	29.84	22	27.73	31.91	−6.36	−2.19	21	0.040	^*∗*^
Be popular	82	46.46	27.23	79	34.94	25.86	48	32.08	26.65	−11.14	−4.38	78	0.000	^*∗∗∗*^	−13.13	−3.89	47	0.000	^*∗∗∗*^	30	47.00	27.18	22	30.00	29.44	−12.27	−2.69	21	0.014	^*∗*^
Be strong	82	39.27	22.98	79	34.94	20.99	48	36.04	19.76	−3.92	−1.85	78	0.068		−0.21	−0.09	47	0.929		30	38.00	17.30	22	35.45	15.95	−4.55	−1.52	21	0.144	
Be on your guard	82	47.44	27.79	79	40.51	27.87	48	36.25	24.20	−6.33	−2.53	78	0.013	^*∗*^	−6.88	−1.79	47	0.081		30	46.67	23.24	22	34.55	24.83	−11.36	−2.49	21	0.021	^*∗*^
I cannot	81	29.38	25.07	79	23.29	23.68	48	17.29	17.95	−6.71	−3.27	78	0.002	^*∗∗*^	−11.46	−4.10	47	0.000	^*∗∗∗*^	29	31.03	22.57	22	22.73	23.54	−7.73	−2.51	21	0.020	^*∗*^
*Ø Stress-amplifying thoughts*	81	39.36	22.72	79	33.59	21.62	48	29.50	18.49	−6.00	−3.10	78	0.003	^*∗∗*^	−7.71	−3.23	47	0.002	^*∗∗*^	29	39.52	18.73	22	30.09	22.06	−8.45	−3.34	21	0.003	^*∗∗*^
Physical warnings	80	31.79	19.38	80	26.43	16.29	48	27.98	16.93	−4.95	−3.27	77	0.002	^*∗∗*^	−3.34	−1.76	46	0.084		29	28.69	16.41	22	24.84	14.31	−4.55	−1.17	21	0.255	
Emotional warnings	81	35.80	25.29	80	25.10	24.82	48	25.69	23.50	−10.23	−4.61	78	0.000	^*∗∗∗*^	−10.82	−3.42	46	0.001	^*∗∗*^	30	43.06	28.03	21	28.97	24.24	−14.68	−2.68	20	0.014	^*∗∗*^
Cognitive warnings	81	29.84	24.15	80	23.13	21.46	48	21.18	16.84	−6.12	−3.19	78	0.002	^*∗∗*^	−7.98	−3.06	46	0.004	^*∗∗*^	30	30.83	23.48	22	28.03	22.94	−5.30	−1.17	21	0.256	
Warnings in behaviour	81	19.68	14.52	80	16.25	11.88	48	17.13	14.17	−3.23	−2.31	78	0.024	^*∗*^	−1.18	−0.56	46	0.579		30	22.41	17.11	22	17.93	13.82	−4.80	−1.21	21	0.241	
*Ø Warning signs for stress*	80	29.13	17.51	80	23.02	15.47	48	23.63	14.75	−5.66	−4.35	77	0.000	^*∗∗∗*^	−4.86	−2.78	46	0.008	^*∗∗*^	29	29.31	16.70	21	24.56	13.12	−6.39	−1.74	20	0.098	

^*∗*^ *p* < 0.05; ^*∗∗*^*p* < 0.01; ^*∗∗∗*^*p* < 0.001.

## Data Availability

Data are available as “hard copy” on paper sheets as well as electronically as SPPS data bank.
